# The Sense of Security and Associated Factors Among Older Home Care Clients: A Register Study

**DOI:** 10.1111/scs.70187

**Published:** 2026-01-15

**Authors:** Palonen Mira, Tuuli Turja, Aaltonen Mari, Edgren Johanna, Kaunonen Marja

**Affiliations:** ^1^ Tampere University Tampere Finland; ^2^ Nursing Research Foundation Helsinki Finland; ^3^ Finnish Institute for Health and Welfare Helsinki Finland

**Keywords:** falls, family support, home care, InterRAI, older people, safety, security

## Abstract

**Aim:**

This study aimed to explore a sense of security and the factors associated with it among older home‐dwelling people who are receiving professional home care.

**Design:**

A cross‐sectional register study.

**Method:**

Register data from the Finnish Institute of Health and Welfare were used for the study. The data were collected between September 2021 and October 2022 from home‐dwelling older adults living in one well‐being service county in Finland and receiving home care services (*N* = 5759). The standardized Resident Assessment Instrument for Home Care (InterRAI‐HC) was used for the data collection. Descriptive statistics and binary logistic regression analysis were applied to identify the determinants of a sense of security.

**Outcome Measures:**

A subjective sense of security.

**Results:**

There was a significant association between a higher subjective sense of security and younger age (aged 85+ OR = 0.982, *p* < 0.001, 95% CI = 0.97–0.99), male gender (OR = 1.866, *p* < 0.001, 95% CI = 1.60–2.18), no deficiencies in housing conditions (OR = 1.224, *p* < 0.001, 95% CI = 1.10–1.37), strong and supportive relationships with family (OR = 1.614, *p* < 0.001, 95% CI = 1.32–1.98), and having had no falls during the past 90 days (OR = 1.351, *p* < 0.001, 95% CI = 1.17–1.56).

**Study Limitations:**

The main limitation of the study is that the register data had been collected by numerous home care professionals and within different home care settings, which may explain the relatively low effect sizes.

**Conclusions:**

Strong family support manifested as the primary societal explanatory factor for a sense of security, whereas home care support services did not play a contributing role. Promoting and enabling good and active family relationships and involving families in the planning of care is a strategy that home health care providers can use to support a sense of security and well‐being among older adults living at home.

## 1 Introduction

The need for safety is one of the key human needs [1]. A sense of security can be defined as an individual's psychological perception of being safe from harm or threat. It can be an objective condition (e.g., low crime rates or environmental hazards) but also a subjective experience that varies between individuals and contexts [1, 2]. In this paper, we focus on the latter. Among older adults, a diminished sense of security may be challenging to verbalize and can present through psychological symptoms such as anxiety, restlessness, and emotional distress. The sense of security among older people is an essential indicator of their quality of life since it is connected to meaningful life experiences and psychological resilience [2]. This is especially relevant among home‐dwelling older people, since maintaining a sense of security is one of the key factors promoting aging in place [3]. Aging in place is a priority in terms of political guidance, and it is what most older people themselves prefer, provided they are not too frail. To that end, most older people live in private households ([4]). Among older people receiving home care services, greater hours of service have been shown to be a central factor in maintaining their sense of security [5].

Although aging in place is a contemporary trend, it has safety concerns. Around half of the EU population of 65+ reports some difficulties in carrying out personal care or household activities, with half reporting a lack of assistance in performing those activities. For 27% of the population, the difficulties were perceived as severe, and of that group, around one‐third used the available home care services [6]. Falls are the most predominant safety issue for home‐dwelling older adults, and globally, more than a third of individuals aged 65+ suffer one or more falls every year; an estimated 37 million falls lead to a need for medical care, and over 680,000 people die as the result of a fall [7].

Falls, or even the mere fear of falling, can have a psychosocial impact on home‐dwelling older people and lead to reduced social contact or the inability to live in place [8]. Together with falls, housing conditions are another safety issue for home‐dwelling older people. In the EU area, around one older person out of every 10 is living in inadequate housing conditions (e.g., with leaks, damp, or rot) [4]. These conditions are viewed as having an indirect impact on quality of life. Deficient living arrangements, such as poor heating or the lack of an elevator, are associated with lower physical functioning and general frailty [9].

For older people, a sense of security includes both social and psychological factors [10]. Security is connected to having trusting relationships with family and friends [2], while family participation is shown to improve the general quality of life of older people [11]. Thus, it is important to consider the meaning of family relationships among older people, including those who are receiving professional home care. Sometimes, professional care and informal care at home are seen as alternatives for older people. However, in addition to professional care, older people frequently receive help from family members that enables them to remain living in their own homes. In addition, family members are key stakeholders in the organization of professional care services. However, it is important to note that family caregivers are often older people themselves, and they may also be frail and burdened by family care responsibilities [12]. Furthermore, not all older people have family support. These perspectives need to be acknowledged when planning policies that aim for safe environments and aging in place.

This study is part of a broader research project “SOL‐TECH: Human‐centered solar smart technology design for healthy aging”, aimed at improving the safety of older home‐dwelling individuals and reducing fall‐related incidents through methods of sustainable development. In this study, we were especially interested to discover which other factors than professional home care are associated with a sense of security among older home‐dwelling people based on register data.

## 2 Aim

The aim of this study was to explore the sense of security among older home‐dwelling people who were receiving professional home care. The aim was explored via research question: Which factors differentiate between a sense of security and its absence among older home care clients?

## 3 Methods

### 3.1 Study Design

A cross‐sectional retrospective register study.

### 3.2 Instrument and Data

The Resident Assessment Instrument (RAI) for home care (interRAI‐HC) was used. The RAI is a standardized tool for assessing clients' service needs and for creating a care, rehabilitation, and service plan for older or disabled persons [13]. According to instructions given by the Finnish Institute for Health and Welfare, home care nurses should assess clients using the RAI twice a year, or when the physician deems it necessary or when the client's situation changes significantly. The assessment should be done either with the home care recipient or with their proxy. In this study, one evaluation per person was used. If there were multiple evaluations during the selected time, the latest evaluation of each person was used.

The interRAI‐HC instrument consists of more than 300 items, covering the physical, cognitive, and psychosocial characteristics of the individual's functioning and information on his/her living environment. It also covers relevant clinical information and has shown substantial reliability in home care settings [14].

The interRAI‐HC data were retrieved from the national RAI database of the Finnish Institute of Health and Welfare (THL) and included a total sample of the latest interRAI‐HC assessment of each home care client aged 65 or more (*n* = 5759) from September 2021 to October 2022 from one well‐being service county in Finland, which consists of approximately 530,000 inhabitants.

The data were used under the Act on the Secondary Use of Health and Social Data [15], and with permission from the Finnish Institute of Health and Welfare (THL/1118/6.02.00/2021).

### 3.3 Measurements

A *subjective sense of security*, as a dependent factor, was measured with a compound variable (*1 = yes*, *0 = no*; Table 1). If the respondent gave an affirmative response to either (1) *a perceived lack of personal security* or (2) *anxiety, restlessness, or distress*, their sense of security was coded as 0. Conversely, a consistently reported sense of security was coded as 1.

**TABLE 1:** Sociodemographic features.

Variable	*n*	%^a^
Age MD = 85 (65–103)		
Gender		
Man	1956	34.0
Woman	3803	66.0
Marital status		
Single	897	15.6
Marriage	1202	20.9
Cohabitation/common‐law marriage/registered relationship	103	1.8
Separation	13	0.2
Widow	2793	48.5
Divorced	750	13.0
Cognitive skills related to daily decision‐making		
Independent	1772	30.8
Almost independent	1695	29.4
Slightly weakened	1257	21.8
Moderately weakened	740	12.8
Significantly weakened	295	5.1
Comprehension		
Understands	2854	49.6
Usually understands	2043	35.5
Often understands	702	12.2
Sometimes understands	138	2.4
Understands rarely or never	22	0.4
Hearing		
Adequate	3270	56.8
Slightly weakened	1545	26.8
Moderately weakened	693	12.0
Significantly weakened	243	4.2
Can't hear	8	0.1
Eyesight		
Adequate	3669	63.7
Slightly weakened	1588	27.6
Moderately weakened	390	6.8
Significantly weakened	97	1.7
Can't see	15	0.3
Form of residence		
Owner‐occupied apartment or rent, no social security services	645	11.2
Owner‐occupied apartment or rental, domestic and/or nursing services	5043	87.6
Assisted living	71	1.2
living arrangements		
Lives alone	4495	78.1
Spouse or partner	980	17.0
Spouse or partner and other(s)	31	0.5
Child(ren) (no partner)	157	2.7
Carer	4	0.1
Siblings	45	0.8
Other relatives	5	0.1
Others than relatives	42	0.7
An accessible apartment		
No	1877	32.6
Yes	3882	67.4
Falls within 90 days		
No	4045	69.9
Yes	1746	30.2
Strong and supportive relationship with family		
Yes	4726	82.1
No	588	10.2
**Sense of security**		
Lack of personal security		
No	5657	98.4
Yes	94	1.6
Self‐evaluated anxiety, restlessness or distress		
No	4380	78.2
Yes	1223	21.8
**Support services** (α.298^b^)		
Meals on wheels		
Weekly	2266	39.3
Less than weekly	3493	60.7
Transport services		
Weekly	504	8.8
Less than weekly	5255	91.2
Receiving volunteer help (other than family)		
Weekly	123	2.1
Less than weekly	5636	97.9
Cleaning service		
Weekly	544	9.4
Less than weekly	5215	90.6
Volunteer escorting to services (other than family)		
Weekly	35	0.6
Less than weekly	5724	99.4
Bathing or sauna service		
Weekly	810	14.1
Less than weekly	4949	85.9
**Housing conditions** (α.421^b^)		
Home in need of renovation		
No	5493	95.5
Yes	259	4.5
Tattered condition – e.g., extremely dirty, rats or vermin		
No	5574	96.9
Yes	180	3.1
Inadequate heating or air conditioning		
No	5511	95.8
Yes	242	4.2
Difficulty getting home/moving around the rooms		
No	5014	87.2
Yes	736	12.8

^a^ Valid percent.

^b^ Cronbach's alpha coefficient.

The independent factors (Table 1) were age, gender, living arrangements (*0 = living alone, 1 = living with a family member*), strong and supportive relationships with family (*1 = yes, 0 = no*), falls (*1 = at least one incident of falling during the past 90 days*, *0 = no falls*), housing conditions (*0 = no deficiency, 1 = deficiency*; 4 items in accordance with the RAI manual, see Table 1), and support services (*1 = weekly, 0 = less than weekly;* 6 items in accordance with the RAI manual, see Table 1). These factors were selected based on the previous theoretical reasoning and based on their statistical association with the binary outcome variable during preliminary testing. The reliability coefficients for the compound items were tested with Cronbach's alpha (see Table 1).

### 3.4 Analysis

Sociodemographic and clinical characteristics data were analyzed using descriptive statistics. Binary logistic regression analysis was used to identify the selected determinants' associations with a sense of security. The results are reported in terms of unstandardized regression coefficients (B), standardized odds ratios (OR), and 95% confidence intervals (CI). The level of significance was established as *p* < 0.001. The data were analyzed using IBMSPSS Statistics for Windows, version 29.0 (IBM Corp., Armonk, New York, USA).

## 4 Results

### 4.1 Sociodemographic Features

The home care service clients were aged between 65 and 103 years (*Md* = 85), and 66% of them were female. The majority of respondents lived alone (*n* = 4495, 78%) or with their spouse or partner (*n* = 980, 17%). Of those who lived alone, the majority were women (*n* = 3133, 69.7%). In total, 1276 people (23%) reported a lack of sense of security. Of them, 4% (*n* = 53) reported a lack of personal security, 93% (*n* = 1182) reported being anxious, restless, or in distress, and 3% (*n* = 41) experienced both. See Table 2.

**TABLE 2:** Binary regression model of factors associated with the sense of security with older home care clients.

	*B*	*p*	OR	95% CI
Aged 85+	−0.018	< 0.001	0.982	0.97–0.99
Male gender	0.624	< 0.001	1.866	1.60–2.18
Living alone	0.207	0.012	1.230	1.05–1.45
No deficiencies in living conditions	0.202	< 0.001	1.224	1.10–1.37
Support services	0.165	0.018	1.179	1.03–1.35
Strong and supportive relationship with family	0.479	< 0.001	1.614	1.32–1.98
No falls during past 90 days	0.301	< 0.001	1.351	1.17–1.56

### 4.2 Explanatory Factors for the Sense of Security Among Older Home Care Clients

There was a significant association between a higher subjective sense of security and younger age (OR = 0.982, *p* < 0.001), male gender (OR = 1.866, *p* < 0.001), no deficiencies in housing conditions (OR = 1.224, *p* < 0.001), strong and supportive relationships with family (OR = 1.614, *p* < 0.001), and having had no falls during the past 90 days (OR = 1.351, *p* < 0.001).

## 5 Discussion

The current study examined, first, the profile of older home care service clients. Second, the study sought to identify the factors that differentiated between having a sense of security and its absence among home care service clients. The average home care service client was found to be female, aged 85 years, and living alone. The key determinants of a subjective sense of security were age, gender, housing conditions, family relations, and a history of falls. Nevertheless, our analysis revealed several explanatory factors that differentiated between individuals who perceived subjective insecurity and those who did not.

In accord with previous findings [16], male gender and age under 85 were associated with a higher sense of security. These results are supported by an earlier study in which female gender and older age were associated with a greater likelihood of frailty [17]. In this study, eight out of ten subjects lived alone, and 70% of them were women. The equivalent situation is found across the EU, with older women more likely to be living alone. In 2018, the proportion of older women living in single person households was 40%, whereas the proportion for older men was 22% [4]. Older home care clients living alone are at greater risk for a low sense of security and a higher proportion of frailty. These factors can further impact their general well‐being and willingness to participate in activities [10]. To mitigate this unfavorable trajectory, social support should be strengthened. Previous studies conducted in Finland and Sweden have reported a 7%–11% proportion of older home‐dwelling people feeling insecurity [2, 10, 18]. Considering the age and gender distribution of the study, we found the fact that fewer than 2% explicitly lacked a sense of personal security in this population surprisingly low. On the other hand, when adding the psychological reactions, the number of people was higher, and it may indicate the difficultness of verbalizing the insecurity.

Supportive family relationships were evaluated as a significant determinant of the higher sense of security reported in this study. This supports earlier findings by Knuutila et al. [10], who found that, in an older population, having children and meeting with friends regularly were associated with a lower likelihood of insecurity. Sincihu et al. [11] also demonstrated the importance of family relationships in securing quality of life. The predictive power of supportive family relationships relative to the difference that living alone or not makes emphasizes the quality of social support over the quantity of social support. This finding supports prior observations that, among the general population, the quality of social support has the most significant impact on the psychological well‐being of individuals [19]. This has also been shown to be true among those who are chronically ill [20]. Although the family importance has been demonstrated also previously (e.g., [21]), our study implies that the quality of social support, that is, family relationships being perceived as strong and supportive, plays a major role as a determinant of a subjective sense of security among older people receiving home care services. To our knowledge, this is the first time the sense of security has been reported as a part of overall service need assessment within the everyday life of older people, rather than among a selected group of participants in a specific research context.

It is noteworthy that, in this study, family support remained meaningful for older people's sense of security in situations where they were also receiving formal home care services. Sometimes, older people are seen as being in an inferior position in these situations [21]. Empowering older people and their families when planning home care can enhance their sense of security and build mutual understanding, as was demonstrated by Sundström et al. [21]. Due to aging in place policies, there is an ever‐growing demand for home care for older individuals. When collaborating with families to provide care in older people's homes, it is crucial to agree on the level of help family members are prepared to commit to, to provide education and support for informal carers, and to ensure that the care does not rely solely on these informal carers [22].

According to the findings, environmental aspects, such as existing housing conditions, also determine the older person's sense of security. This is to be expected, because older people typically spend most of the day inside the house [23, 24]. Thus, the home should be safe and in sufficiently good condition to support an overall feeling of security. In Chang et al. [25], housing conditions were the strongest predictor of place dependence, forming one dimension of place attachment. Furthermore, in an earlier study focusing on evenings and nighttime, a familiar environment and the prevention of falls were both significant factors in enabling older people receiving home care to continue living in an ordinary home [26]. In this study, the environmental aspect focused on housing conditions. In future studies, the focus could be on the broader environmental context, such as the living area.

Based on the results of both this and earlier studies (see [27]), fall prevention is a crucial yet difficult element to control when promoting safety and aging in place.

Globally, numerous national strategies have aimed to improve this area. In Finland, national recommendations by the Finnish Physiotherapy Society [28] for the prevention of falls and fall‐related traumas highlight the importance of identifying persons at risk of falling and of planning and implementing prevention measures, such as individually planned and implemented housing and living environment modifications. However, to prevent falls and fall‐related injuries in older people living at home, versatile physical exercise is essential to maintain adequate physical functioning and mobility [29]. For lasting solutions, sustainable product development and participatory co‐design elements are crucial. One example of such an element is the project under which this paper has been conducted, called SOL‐TECH: Human‐centered solar smart technology design for healthy aging (see [30]).

Through an evaluation of these determinants of a sense of security, it is possible to plan individual support services to enable older people to stay in their homes and live independently for as long as they find it meaningful.

### 5.1 Study Strengths and Limitations

This study used data from a population‐based register providing information about home care recipients in one selected well‐being service county in Finland. Statistical significance is reported in this study as indicating the generalizability of the findings. However, statistical significance is less relevant for reporting the parameters of the population sample.

Population‐based data are considered less biased than sampling. Nevertheless, the RAI data have some reliability issues. This study used data from one well‐being service county. This area was selected for this sub‐study in accordance with the overall aim of the research project SOL‐TECH. This may have led to some bias between well‐being service counties. However, the data were representative of the target population within this area. Also, the data were collected by numerous home care professionals in different home care settings, and this can explain the relatively low effect sizes reported in this paper. Also, some assessments were delivered by a care provider proxy, and this may represent a source of respondent bias [31]. However, the RAI assessments were largely collected in collaboration with the residents themselves, which indicates higher reliability compared to data collection from proxies only.

One limitation of the study was that the measurements of sense of security were limited to the perceived lack of personal security and/or anxiety, restlessness, or distress. With for example, qualitative approach, other aspects of the phenomenon could have been explored, but in this case, the study was limited by variables available in the register data. Also, the Cronbach's alpha coefficients for these compound variables remained low, despite the item‐level analysis. However, the items were combined in accordance with the RAI instrument manual. After consideration, we decided to use those items in this study, as these variables are central from a content‐related perspective.

As a further method‐related limitation, the cross‐sectional study design lacks causal evidence, and future longitudinal studies are recommended.

## 6 Conclusion

A sense of security among older home care clients is determined by personal, societal, and environmental factors. Our analysis showed that male gender increases the odds of a subjective sense of security by nearly two times. As a primary societal explanatory factor, people with strong family support are more likely to experience a subjective sense of security. In contrast, home care support services do not seem to make a major contribution to a subjective sense of security. This study suggests that promoting and enabling good and active family relationships and involving the family in care planning is a strategy that home health care providers can use to support a sense of security and well‐being among older adults living at home.

## Author Contributions

**Palonen Mira:** conceptualisation, methodology, data processing, analysis, writing the original draft. **Tuuli Turja:** conceptualisation, methodology, analysis, review and editing. **Aaltonen Mari:** conceptualisation, analysis, review and editing. **Edgren Johanna** and **Kaunonen Marja:** conceptualisation, review and editing. All authors approved the final version.

## Funding

This study was funded by the Jane and Aatos Erkko Foundation, Finland.

## Ethics Statement

The Institutional Review Board (IRB) of the Finnish Institute for Health and Welfare reviewed and approved the study (11/2011§419‐§430), and the research was conducted with permission from the Finnish Institute for Health and Welfare (THL/1118/6.02.00/2021). No data other than retrospective registry data were used in this study; thus, the need for written informed consent was waived by the IRB due to the retrospective nature of the study and based on the Act on Data Protection (2018/1050) and the Act on the Finnish Institute of Health and Welfare (668/2008).

## Conflicts of Interest

The authors declare no conflicts of interest.

## Data Availability

The data are not publicly available. For this study, data were made available under the Act on the Secondary Use of Health and Social Data [15], and with permission from the Finnish Institute of Health and Welfare (THL/1118/6.02.00/2021).
